# Comprehensive analysis of ZNF family genes in prognosis, immunity, and treatment of esophageal cancer

**DOI:** 10.1186/s12885-023-10779-5

**Published:** 2023-04-03

**Authors:** Kunqiao Hong, Qian Yang, Haisen Yin, Na Wei, Wei Wang, Baoping Yu

**Affiliations:** 1 Department of Gastroenterology, Renmin Hospital of Wuhan University, Wuhan, China; 2 Key Laboratory of Hubei Province for Digestive System Disease, Wuhan, China; 3 Central Laboratory, Renmin Hospital of Wuhan University, Wuhan, China; 4 Department of Gastroenterology, Guizhou Provincial People’s Hospital, Guiyang City, Guizhou province China; 5 NHC key Laboratory of Pulmonary Immune-related Disease, Guizhou Provincial People’s Hospital, Guiyang City, Guizhou province China; 6 Department of Gastroenterology, Affiliated Hospital of Hubei, Xiangyang Central Hospital, University of Arts and Science, Hubei, China

**Keywords:** Esophageal cancer, ZNF family genes, Prognosis, Risk model, Nomogram

## Abstract

**Background:**

As a common malignant tumor, esophageal carcinoma (ESCA) has a low early diagnosis rate and poor prognosis. This study aimed to construct the prognostic features composed of ZNF family genes to effectively predict the prognosis of ESCA patients.

**Methods:**

The mRNA expression matrix and clinical data were downloaded from TCGA and GEO database. Using univariate Cox analysis, lasso regression and multivariate Cox analysis, we screened six prognosis-related ZNF family genes to construct the prognostic model. We then used Kaplan-Meier plot, time-dependent receiver operating characteristic (ROC), multivariable Cox regression analysis of clinical information, and nomogram to evaluate the prognostic value within and across sets, separately and combined. We also validated the prognostic value of the six-gene signature using GSE53624 dataset. The different immune status was observed in the single sample Gene Set Enrichment Analysis (ssGSEA). Finally, real-time quantitative PCR was used to detect the expression of six prognostic ZNF genes in twelve pairs of ESCA and adjacent normal tissues.

**Results:**

A six prognosis-related ZNF family genes model consisted of ZNF91, ZNF586, ZNF502, ZNF865, ZNF106 and ZNF225 was identified. Multivariable Cox regression analysis revealed that six prognosis-related ZNF family genes were independent prognostic factors for overall survival of ESCA patients in TCGA and GSE53624. Further, a prognostic nomogram including the riskScore, age, gender, T, stage was constructed, and TCGA/GSE53624-based calibration plots indicated its excellent predictive performance. Drug Sensitivity and ssGSEA analysis showed that the six genes model was closely related to immune cells infiltration and could be used as a potential predictor of chemotherapy sensitivity.

**Conclusion:**

We identified six prognosis-related ZNF family genes model of ESCA, which provide evidence for individualized prevention and treatment.

**Supplementary Information:**

The online version contains supplementary material available at 10.1186/s12885-023-10779-5.

## Introduction

Esophageal cancer (ESCA) ranks seventh for incidence (604,000) and sixth for mortality (544,000), which means it is responsible for one in eighteen cancer deaths [1]. Traditional prognostic methods, such as histopathology and tumor staging systems, are of limited use and early detection remains a difficult goal [2]. Due to the lack of specific methods for early diagnosis and treatment, ESCA patients’ five-year survival rate is remains dismal [3]. The poor outcome urges to identify robust biomarkers for predicting the prognosis of ESCA patients.

Zinc finger protein encoded by nearly 5% of the human genome is the largest family of transcription factor proteins, which has finger-like DNA binding do-mains and plays an important role in many biological processes [4]. So far, zinc finger motifs have been classified into eight different classes based on their mainchain conformations and secondary structures around zinc-binding sites, including Cys2his2(C2H2)-like, ZN2/Cys6, Treble clef, zinc band, Gag joint, Taz2 domain-like, zinc-binding ring and metallothionein [5]. Due to the diversity of zinc finger motifs and these domains, ZFPs play different gene regulatory roles in different cellular environments and stimuli. ZNF306 promotes the development of colorectal cancer by transcriptionally activating integrin β4 and vascular endothelial growth factor [6]. ZNF384 promotes the proliferation of Hepatocellular carcinoma by directly up-regulating the expression of cyclin D1 [7]. Upregulation of ZNF554 is a potential tumor suppressor and its decreased expression may lead to the loss of oncogene suppression, activation of tumor pathways, and shorter survival of patients with malignant glioma [8]. The overexpression of ZNF655 promoted the progression of glioma by binding to the promoter of AURKA [9]. ZNF410 represents a special class of gene regulators, a conserved TF, which has a unique regulatory role on chromatin subcomplexes [10].Taken together, these studies indicate that ZNF genes may function as oncogenes involved in the occurrence and progression of cancer.

With the development of large-scale genome sequencing technologies, the integration of prognostic-related genetic markers has improved the level of early diagnosis of cancer compared with traditional clinical parameters. In the current study, we screened prognostic-associated ZNF family genes from TCGA dataset and validated the prognostic value of the six-gene signature using GEO dataset. We also constructed a nomogram based on the riskScore and clinical characteristics to predict individual overall survival (OS). In conclusion, our work may contribute to the early diagnosis of ESCA patients.

## Materials and methods

### Collection of data

RNA sequencing data of ESCA patients were extracted from the Cancer Genome Atlas (TCGA, https://cancergenome.nih.gov) and Gene Expression Omnibus (GEO, https://www.ncbi.nlm.nih.gov/geo/). Relevant clinical information, including age, gender grade, survival status, and TNM stage, were also acquired. In this study, we collected 163 ESCA samples and 11 adjacent normal samples from TCGA and 119 ESCA samples from GSE53624 dataset. It has become evident that normalization of RNA-Seq data is necessary for reliable inferences and replication of results [11] [12]. GEO data is in TPM format, whereas TCGA data is in FPKM format. In this study, we first transform TCGA data into TPM format, then normalize TCGA and GEO data using the SVA package to minimize the batch effect.The expression levels of ZNF family genes were extracted for survival analysis, and the prognosis-related ZNF genes were identified. The prognostic model was constructed by using these genes, then verify the accuracy of the model.

### Establishment and testing of the risk score model

We randomly separated the TCGA-ESCA patient samples into training and testing groups. The training cohort was used to establish construct the prognosis model and the testing cohort was used to verify the model [13]. By applying differential expression analysis, univariate Cox regression analysis, least absolute shrinkage and selection operator (LASSO) regression analysis and multivariate Cox regression analysis, we identified six prognostic related ZNF family genes which can be viewed as a signature to predict the disease outcomes of patients with ESCA. Subsequently, we divided the training group into high- and low-risk groups using the median risk score as the cut-off point. Kaplan–Meier survival curve analysis was performed to show the difference in OS between the two groups. We also plotted the time-dependent receiver operating characteristic (ROC) curve and risk nomogram to evaluate the prediction accuracy of the model.

Furthermore, univariate and multivariate Cox analyses were performed to confirm that the six prognostic-related genes signature was an independent prognostic factor for ESCA compared to other clinical characteristics—such as age, sex, clinical stage, TNM, and risk score. We analyzed the correlation between the risk scores and clinical parameters to investigate whether there was any difference in risk scores among different clinical parameter stratification.

### Gene-set enrichment analysis

Gene ontology (GO) term enrichment analysis and Kyoto Encyclopedia of Genes and Genomes (KEGG) pathway analysis were performed to investigate potential signaling pathways and functions related to the six ZNF family genes included in the model [14].

### Immune infiltration analysis and prediction of the sensitivity toward chemotherapeutic agents

We used the R package “GSEABase” to investigate the differential expression and function of 23 infiltrating immune cells between the high- and low-risk groups. We also used the R package “pRRophetic” to analyze drug sensitivity in the high- and low-risk groups.

### Quantitative real-time PCR

Total RNA was extracted using Trizol (Cat#9109; TaKaRa, Japan) and reversed into cDNA by PrimeScript RT Master Mix kit (RR047A; Takara, Japan). All mRNA levels were assessed using the SYBR Green PCR Mix (RR420A; Takara, Japan) and the CFX Connect (BIO-RAD, USA). All experiments were performed in triplicate and analyzed with the 2^−ΔΔ^CT method. Supplementary Table 1 lists the primer sequences.

### Statistical analysis

All data were expressed as the mean and standard deviation of at least three independent experiments and analyzed by GraphPad Prism 7.0 software (La Jolla, CA, USA). R software (version x64 4.0.5) was used for statistical analysis, including Cox regression analysis, ROC curve analysis, gene enrichment analysis, and immune infiltration analysis.

## Results

### Features of patients with ESCA enrolled in this study

Sequencing data and corresponding clinical data of ESCA, including 163 tumors and 11 paracancerous tissues, were obtained from the TCGA database. Clinical information of 163 ESCA patients including survival status, grade of tumor, American Joint Committee on Cancer (AJCC) stage, classification of tumor, lymph node, and metastasis was summarized in Supplementary Table 2.

### Construction and evaluation of the prognostic risk signature

**Fig. 1:**
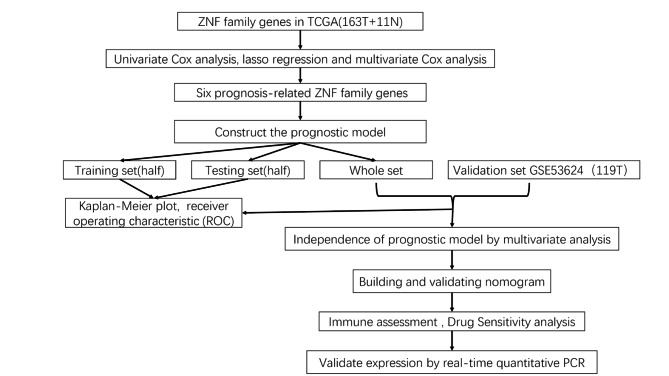
Flowchart for generating and validating the six prognosis-related ZNF family genes signature

**Fig. 2:**
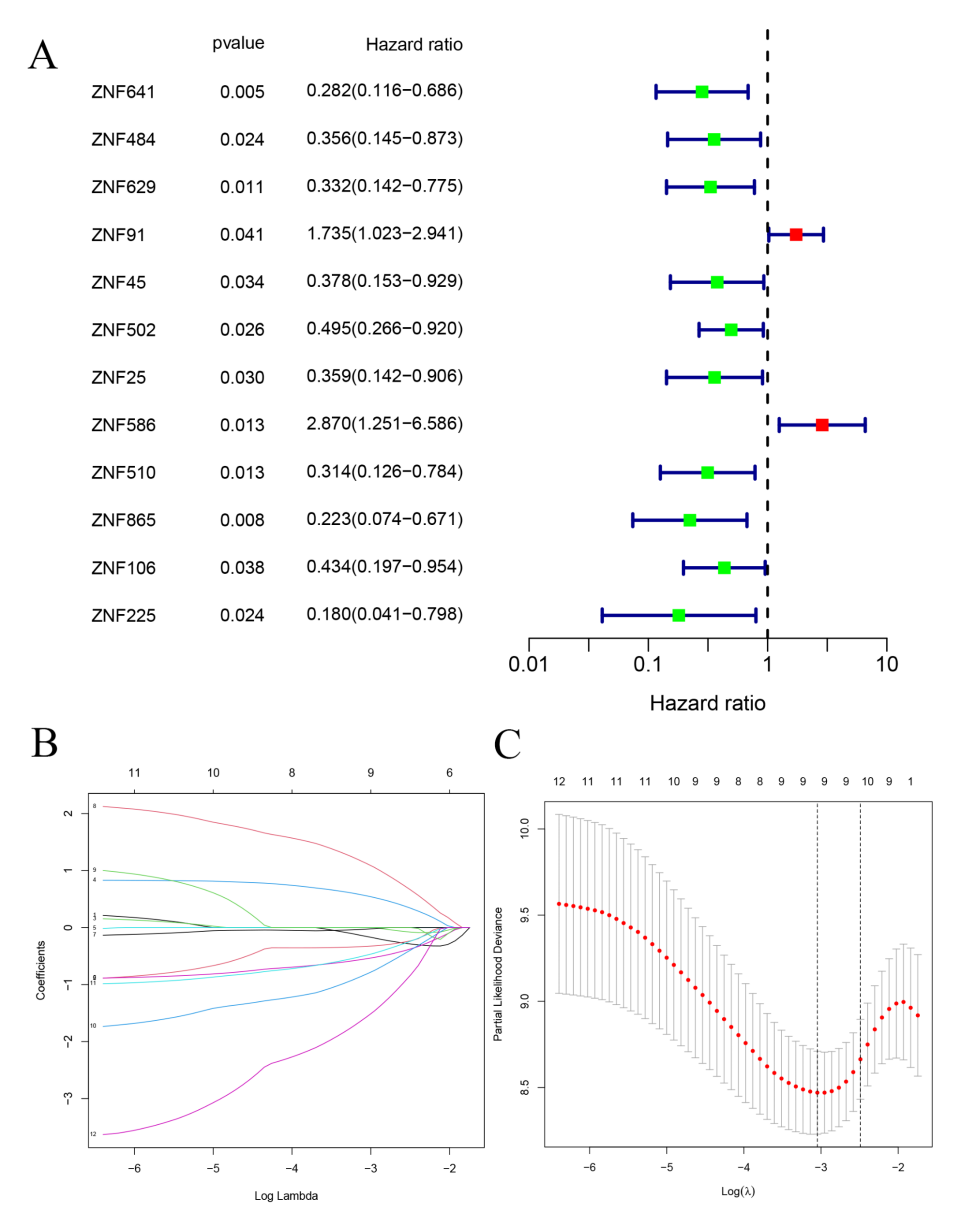
Selection of prognostic ZNF family genes with prognostic value. (**A**) Risk ratio forest plot shows that twelve prognosis-related ZNF family genes, were significantly related to OS of ESCA patients. (**B**) Adjusted parameters of LASSO regression model. (**C**) Figure for LASSO coefficient spectrum of prognostic ZNF family genes

**Fig. 3:**
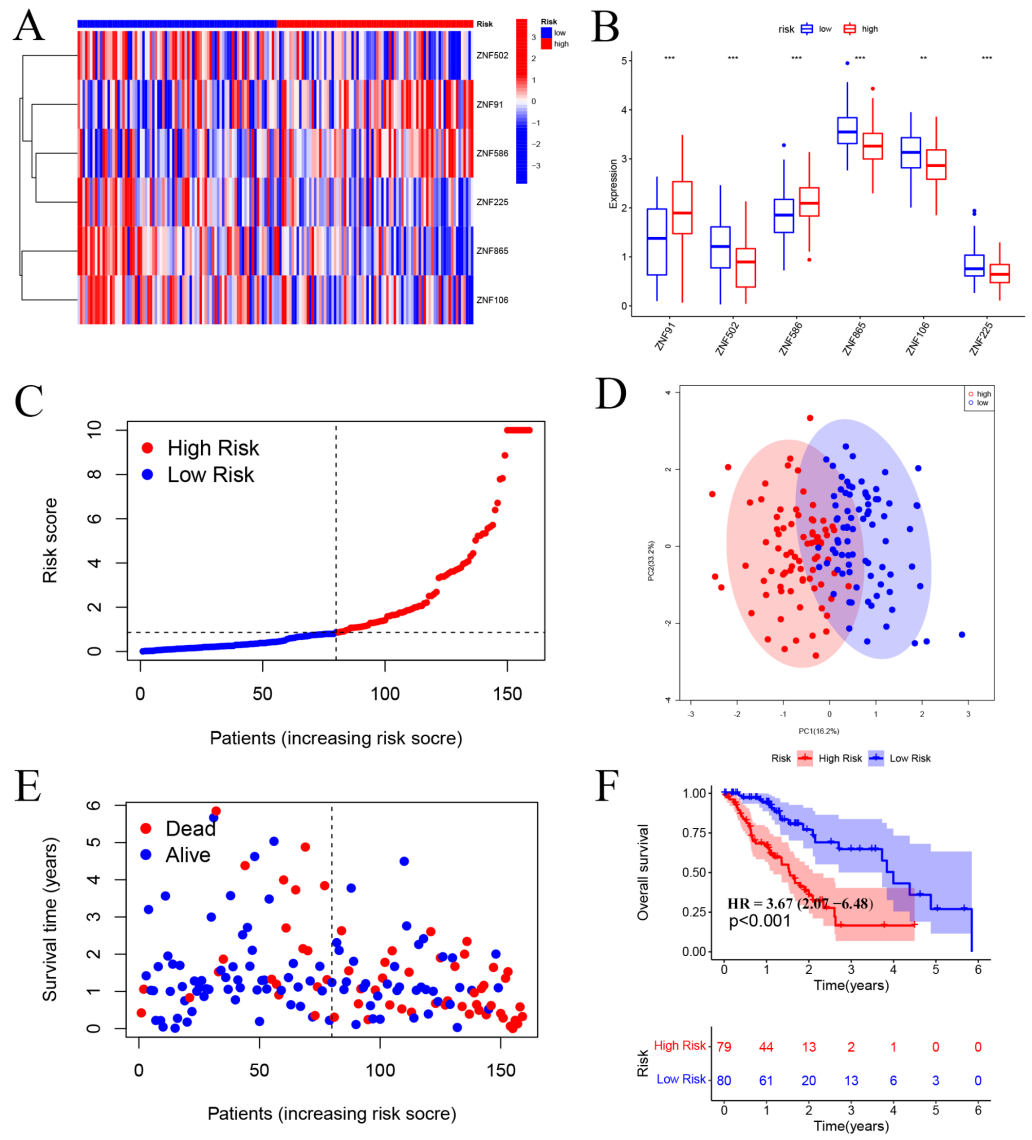
ZNF family genes signature predicts overall survival in patients with ESCA. (**A-B**) A heatmap and box plot showed the differential expression of six prognosis-related ZNF family genes between high-and low-risk subgroups. The gene expression was scaled by log_2_ (original expression of gene + 1). (**C-D**) The distribution of risk scores for each patient. With the median risk score as the cutoff, ESCA patients were divided into high- and low-risk subgroups. (**E**) Relationship between survival time (years) and survival status for each patient. (**F**) Kaplan-Meier curve of patients in the high- and low-risk subgroups to validate the predictive value of ZNF genes signature. The difference between the high- and low-risk subgroups was measured by the log-rank test, with a *P*-value < 0.001. OS, Overall Survival

This study was designed to investigate the prognostic significance of ZNF family in ESCA. The process of constructing and validating the six prognosis-related ZNF family genes signature is shown in Fig. 1. First, we performed univariate Cox regression and identified 12 remarkably prognosis-associated gens (ZNF641, ZNF484, ZNF629, ZNF91, ZNF45, ZNF502, ZNF25, ZNF586, ZNF510, ZNF865, ZNF106, and ZNF225) (Fig. 2A). Then, we used LASSO regression to build the prognostic signature and determine the coefficients (Fig. 2B -C). Finally, six ZNF family genes were enrolled in the signature, and each coefficient represented the weight of the expression of the corresponding coefficients (*P* < 0.05, Supplementary Table 3). The unpaired *t*-test was used to analyze the expression differences of six prognosis-related ZNF family genes in 163 ESCA patients and 11 normal esophageal samples. These findings suggested that compared with normal esophageal tissues, the expression of ZNF91 and ZNF586 were upregulated, while ZNF502, ZNF865, ZNF106, ZNF225 were downregulated in ESCA patients (Fig. 3A-B).

Next, we used multivariate Cox regression to calculate their respective coefficients (βi) to establish a risk score model. We set the median risk.

score as the cutoff value and divided 173 patients into high-risk and low-risk groups (Fig. 3C). Then, we performed PCA to assess the distinct distribution between the high- and low-risk groups. Patients tended to separate into two clusters, which clearly indicated that the status of ESCA patients in the two risk score groups was different (Fig. 3D). As shown in the scatter plot, The higher the risk the more death patients (Fig. 3E). Additionally, the negative correlation between risk score and prognosis was affirmed by Kaplan-Meier survival curve (*P* < 0.001, Fig. 3F).

### Testing of the risk score model

**Fig. 4:**
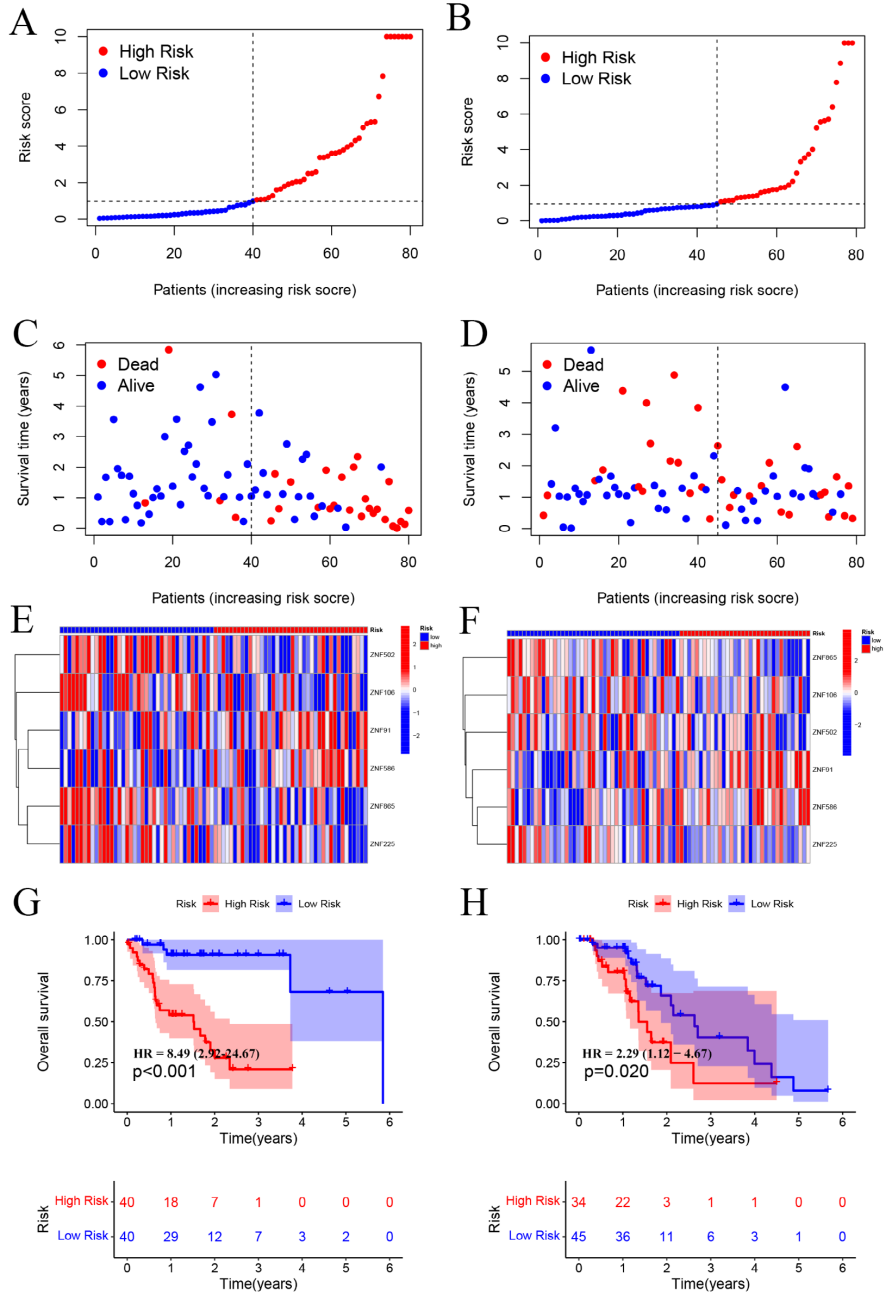
Development and validation of the risk model for patients with ESCA. (**A, B**) Distribution of the ESCA patients with different risk scores in the training set and testing set. According to the median of the patient’s risk score, the ESCA patients were divided into high- (red) and low-risk(blue) groups. (**C, D**) The distribution of survival status of ESCA patients. The blue blots represent the patients who are alive, and red represents the patients who are dead. (**E, F**) Heat map depicting the expression patterns in the six prognosis-related ZNF family genes between high- and low-risk groups. (**G, H**) Overall survival (OS) of high-risk and low-risk patients in the testing group and training group

After calculating the risk scores of all patients in TCGA, we divided the training set and testing set samples into high- and low-risk groups according to the median value of risk score, as shown in Fig. 4A and B. We found that in both the training and testing sets, the proportion of patients with ESCA who died in the high-risk group was higher than that in the low-risk group (Fig. 4C-D). Moreover, six prognosis-related ZNF family genes in the risk model showed the same expression pattern in the training and testing sets (Fig. 4E-F). The Kaplan-Meier survival curve showed that the clinical outcomes of patients in the low-risk group were better than those in the high-risk group (*P* = 0.001; Fig. 4G), both in the testing set (*P* = 0.02; Fig. 4H).

### Correlation between the model and the clinical parameters

**Fig. 5:**
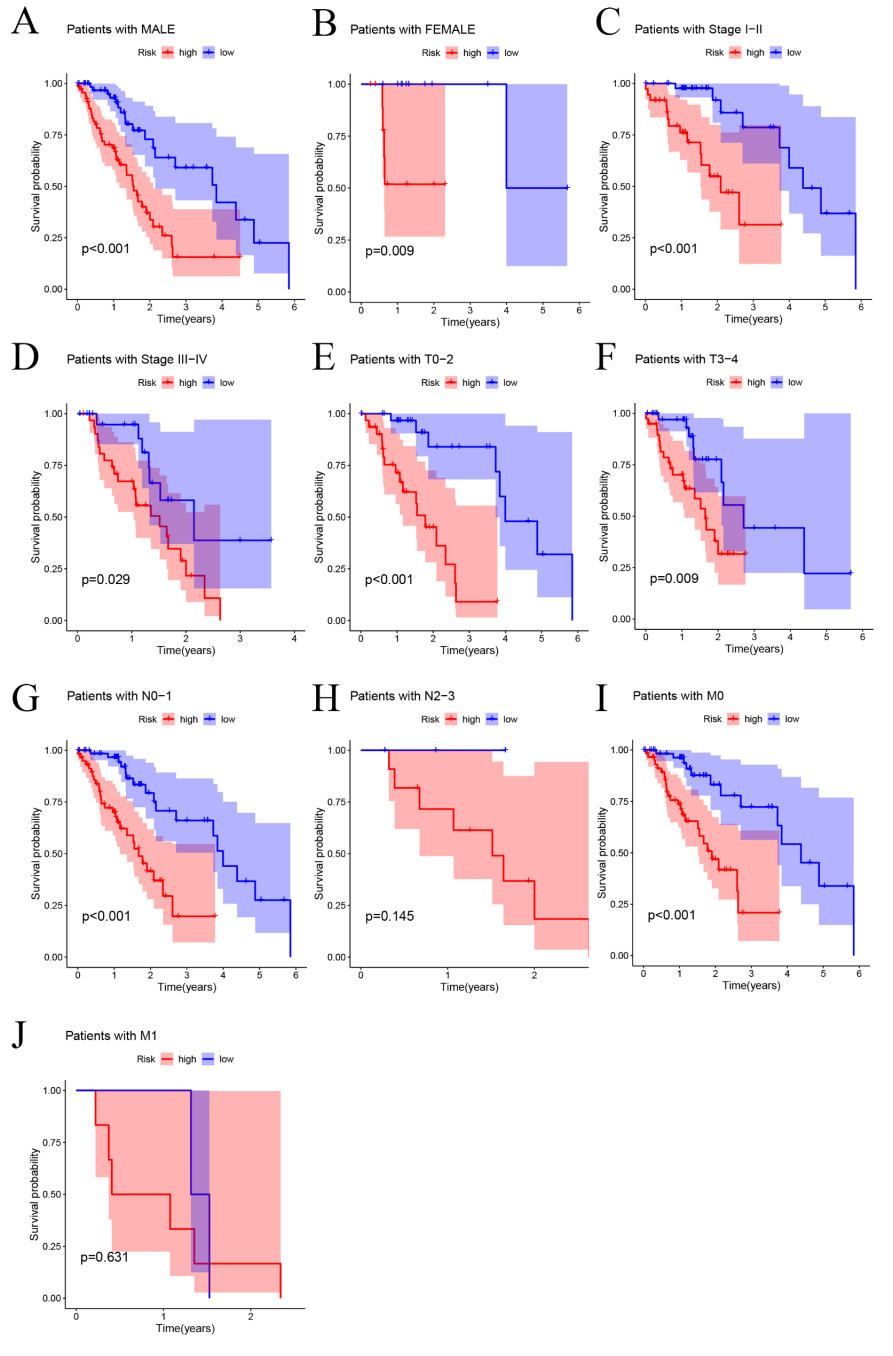
Kaplan-Meier curves showing the differences in prognosis between the high- and low- risk groups in different clinical subgroups, including male (**A**), female (**B**), stage I-II (**C**), stage III-IV (**D**), T0-2 (**E**), T3-4 (**F**), N0-1 (**G**), N2-3 (**H**), M0(**I**), M1(**J**)

We then analyzed the correlation between the risk scores and other clinical parameters. According to the Kaplan-Meier analysis, the model based on six prognosis-related ZNF family genes had significantly distinct risk stratification ability in ESCA. The results presented that the high-risk patients in ESCA exhibited obviously worse prognosis (Fig. 5A-G, I). Whereas, in the patients with N2-3 (Fig. 5H), M1 (Fig. 5J) subgroups, this conclusion did not hold.

### Independent prognostic analysis and construction of a nomogram

**Fig. 6:**
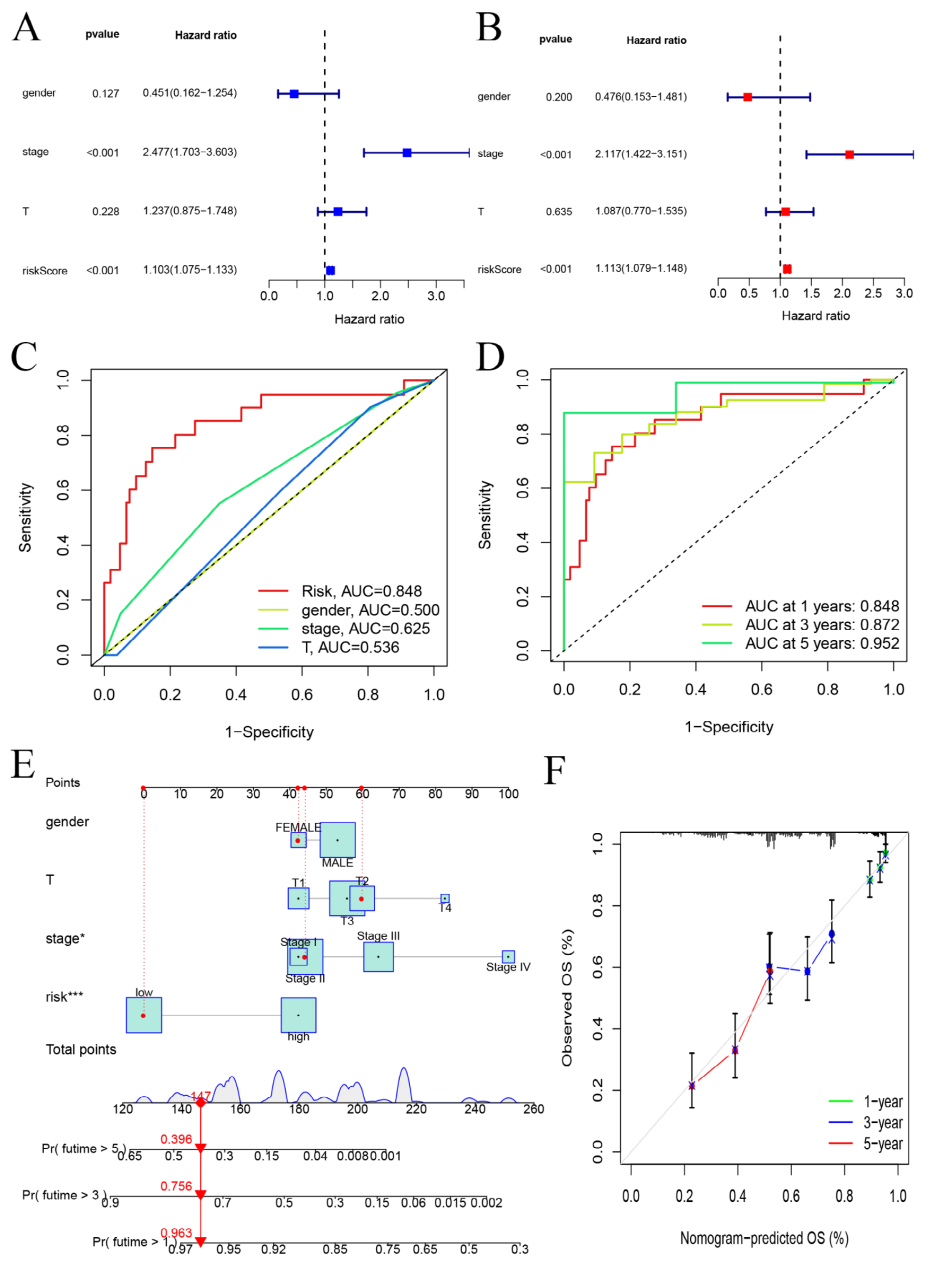
Assessing risk factors and constructing nomogram of prognosis. Univariate analysis (**A**) and multivariate analysis (**B**) were performed for screening of risk factors. (**C**) The ROC curves of clinicopathological characteristics and risk score for 1-year OS. (**D**) The ROC curves for 1-, 3-, and 5-year OS (**E**) An established nomogram model incorporated with the six prognosis-related ZNF family genes and clinicopathological parameters for prediction of OS in the TCGA dataset. (**F**) Calibration curves showed the concordances between predicted and observed 1-, 3-, and 5-year survival rates of ESCA patients based on the nomogram after bias corrections

Both univariate and multivariate Cox analyses showed gender, stage, T grade and the prognostic risk model could be used independently to predict the prognosis of ESCA (Fig. 6A-B). We then further compared these variables and found that the risk score was more accurate than the pathological stage and age in predicting OS at one years. The AUCs at one years for the risk score, gender, stage and the T grade were 0.848, 0.500, 0.625 and 0.536, respectively (Fig. 6C). We also drew a time-dependent ROC curve for the patients in the two groups (Fig. 6D). The AUC values for the risk score at 1, 3 and 5 years were 0.848, 0.872, 0.952. We also constructed a nomogram to estimate the probability of survival at 1, 3 and 5 years. The predictive factors including gender, stage, T stage, and the ZNF family genes prognostic signature, were used to construct the nomogram for OS (Fig. 6E). The C-index value of the nomogram was 0.826. The calibration curves depicting the actual and nomogram-predicted survival at 1, 3 and 5 years were relatively in accord with the reference lines (Fig. 6F). These results suggest that the nomogram including our prognostic signature is precise and reliable.

### External validation of the prognostic gene signature

**Fig. 7:**
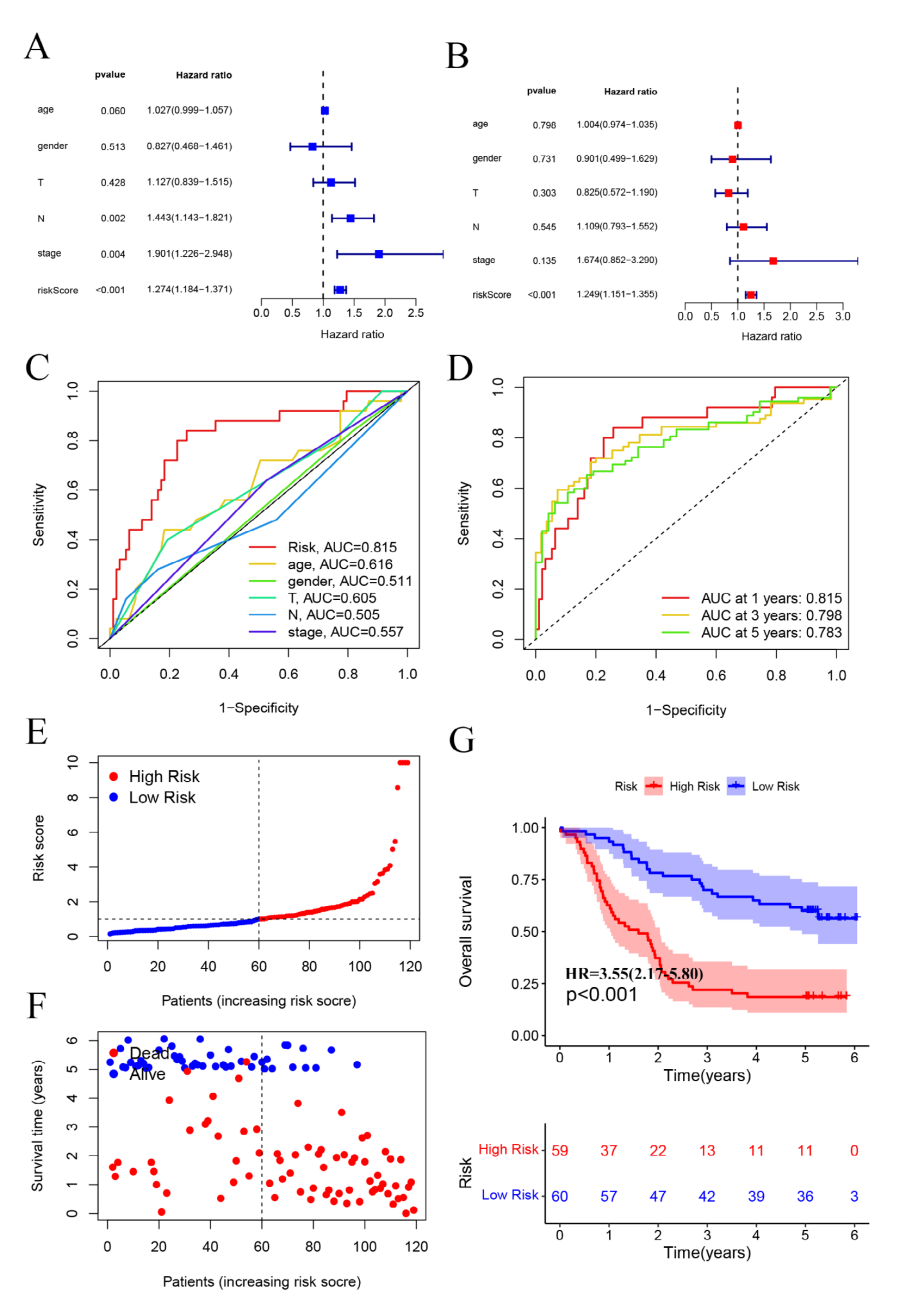
External validation of the prognostic gene signature. Univariate analysis (**A**) and multivariate analysis (**B**) were performed for screening of risk factors in GEO dataset. (**C**) The ROC curves of clinicopathological characteristics and risk score for 1-year OS. (**D**) The ROC curves for 1-, 3-, and 5-year OS. (**E**) Distribution of the ESCA patients with different risk scores in high- and low riskScore groups. (**F**) The distribution of survival status of ESCA patients. (**G**) Overall survival (OS) of high-risk and low-risk patients

**Fig. 8:**
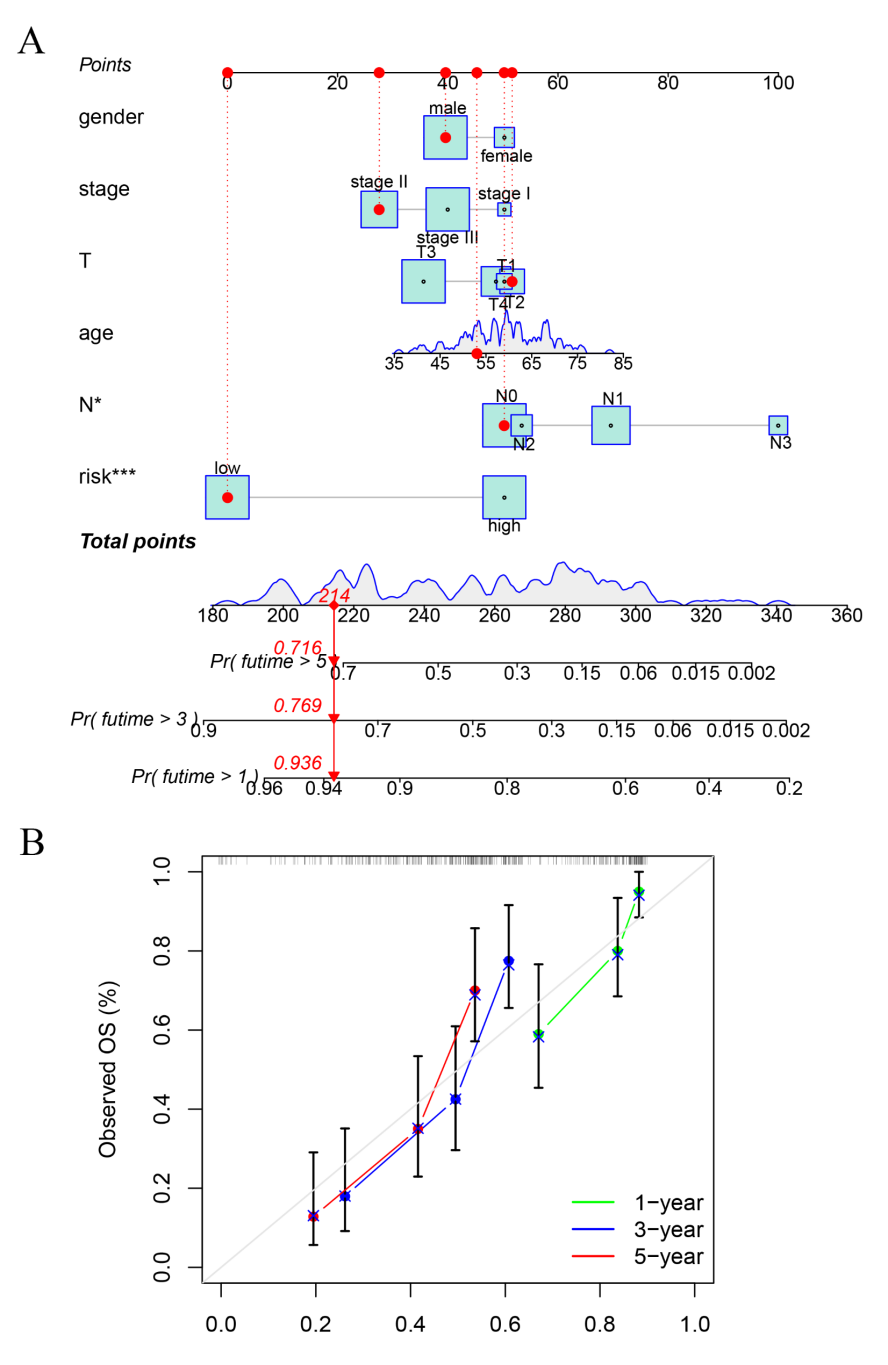
Established nomogram model. (**A**) An established nomogram model incorporated with the six prognosis-related ZNF family genes and clinicopathological parameters for prediction of OS in the GEO dataset. (**B**) Calibration curves showed the concordances between predicted and observed 1-, 3-, and 5-year survival rates of ESCA patients based on the nomogram after bias corrections

To confirm the prognostic model had similar predictive values in different populations, the GEO cohort was used for external confirmation. Supplementary Table 2 shows the demographics and clinicopathologic characteristics of ESCA patients in the GEO validation cohort. Similarly, we performed univariate (riskScore (*P* < 0.001)) and multivariate Cox regression analyses (riskScore (*P* < 0.001)) to evaluate the prognostic significance of the model combined with various clinicopathologic parameters (Fig. 7A-B). In addition, risk score was more accurate than the clinicopathologic parameters in predicting OS at one years. The AUCs at one years for the riskScore, age, gender, T, N, and stage were 0.815, 0.616, 0.511, 0.605, 0.505 and 0.557, respectively (Fig. 7C). Therefore, the prognostic riskScore model constructed by the GEO validation cohorts was an independent prognostic factor for ESCA. The riskScore model also showed a favorable predictive ability for the 1-, 3- and 5-year OS rates, with AUC values of 0.815, 0.798 and 0.783, respectively (Fig. 7D). Furthermore, total of 119 ESCA patients in the GEO set were classified into low- and high-risk group (Fig. 7E-F), and the OS of the ESCA patients in the high-risk group was significantly lower than that of the patients in the low-risk group (*P* < 0.001; Fig. 7G). Finally, we established a prognostic nomogram to predict the survival probability based on the GEO validation cohort (Fig. 8A). The calibration curves depicting the actual and nomogram-predicted survival at 1, 3 and 5 years were relatively in accord with the reference lines (Fig. 8B).

### Functional enrichment analysis and evaluation of immune cell infiltration

**Fig. 9:**
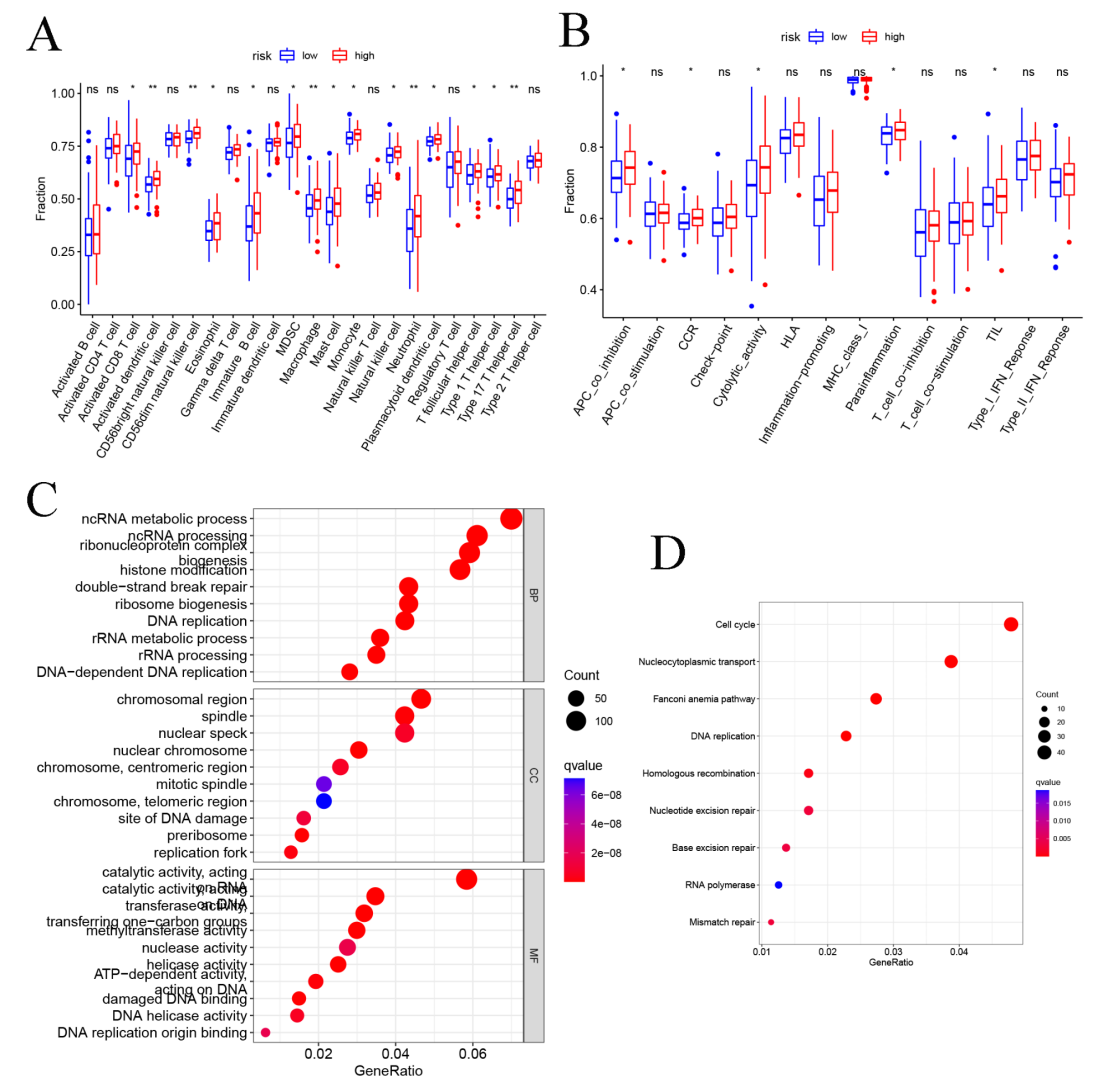
Functional enrichment analysis and immune analysis. (**A**) Comparison of the infiltration of 21 immune cells between the different risk-groups. (**B**) Comparison of 14 immune-related functions between the different risk-groups. (**C**) GO analysis of highly related mRNAs. (**D**) KEGG analysis of highly related mRNAs. BP: biological process. CC: cellular component. MF: molecular function. *P < 0.05, **P < 0.01, ***P < 0.001

To evaluate the infiltration scores of immune cells and immune-related functions, we performed ssGSEA analysis to quantify the scores of immune cell infiltration and immunity-related functions. In the high-risk patients, activated dendritic cell, CD56dim natural killer cell, macrophage, neutrophil and type 17 T helper cell were significantly higher than in low-risk patients (*P* < 0.01; Fig. 9A). In the high-risk patients, the functions were at higher levels, including ABC-co-inhibition, CCR, cytolytic activity, parainflammation, and TIL (*P* < 0.05; Fig. 9B). Our investigation indicated that the high-risk group had elevated immune activity, which might contribute to the occurrence of cancer. We performed GO analysis of the mRNAs co-expressed with the six prognosis-related ZNF family genes. GO and KEGG enrichment analysis showed that the target genes were mainly enriched in cell cycle, DNA replication and Fanconi anemia pathway (Fig. 9C-D).

### Analysis of drug sensitivity

**Fig. 10:**
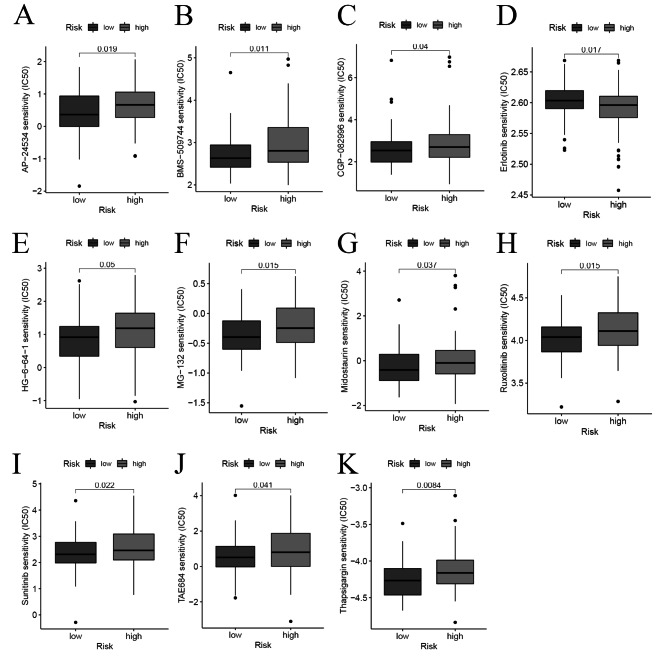
Drug sensitivity analysis to drugs of high- and low-risk subgroups. Differential chemotherapeutic responses in high- and low-risk patients (**A-K**).

We analyzed the sensitivity difference of chemotherapy drugs for ESCA in the current stage of clinical trials between the high- and low-risk groups, with the drug sensitivity expressed by IC50. We showed that patients in the high-risk group were more sensitive to AP-24,534 (*P* = 0.019, Fig. 10A), BMS-509,744 (*P* = 0.011, Fig. 10B), CGP-082996 (*P* = 0.04, Fig. 10C), HG-6-64-1 (*P* = 0.05, Fig. 10E), MG-132 (*P* = 0.015, Fig. 10F), Midostaurin (*P* = 0.037 Fig. 10G,), Ruxolitinib (*P* = 0.015 Fig. 10H,) Sunitinib (*P* = 0.022, Fig. 10I), TAE684 (*P* = 0.041, Fig. 10J), and Thapsigargin (*P* = 0.0084, Fig. 10K), whereas patients in low-risk group were more sensitive to Erlotinib (*P* = 0.017, Fig. 10D), which indicated that the model could be used as a potential predictor of chemotherapy sensitivity.

### External validation in expression

**Fig. 11:**
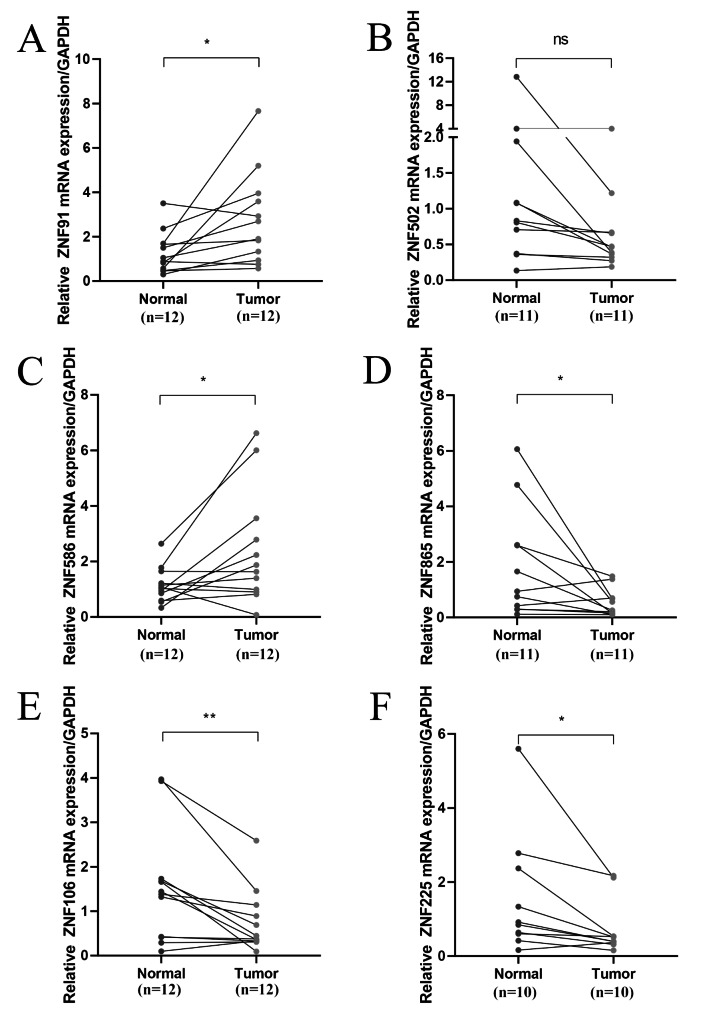
Expression of six prognosis-related ZNF family genes in twelve pairs of esophageal cancer tissue samples. *P < 0.05; **P < 0.01; ns > 0.05

RT-qPCR assay was conducted to quantify the mRNA level of the six ZNF family genes in 12 pairs of esophageal cancer tissue samples (Fig. 11). Results indicate that ZNF91 (Fig. 11A), and ZNF586 (Fig. 11C) were upregulated in esophageal cancer tissue than adjacent tissue, whereas ZNF502 (Fig. 11B), ZNF865 (Fig. 11D), ZNF106 (Fig. 11E), and ZNF225 (Fig. 11F) was downregulated.

## Discussion

Esophageal cancer is one of the most common cancers with high morbidity and mortality [15]. Current therapeutic strategies for ESCA include surgery, chemotherapy, radiotherapy, molecular targeted therapy and their combination [16, 17]. In addition, immunotherapy is also playing an increasingly significant role [2, 18]. However, the prognosis remains poor and the overall Five-year survival rate is very low [19]. Therefore, achieving early diagnosis and effective treatment remains challenging. Identification of novel biomarkers will help assess prognosis, screen out patients in need of Immune intervention and drug therapy. Zinc finger proteins (ZFPs) primarily function as transcription factors in tumorigenesis and tumor progression involved in various tumor, such as esophageal squamous cell carcinoma cells [20],lung cancer [21], hepatocellular carcinoma [7], kidney renal clear cell carcinoma [22], oral squamous cell carcinoma [23]. Transcription factors (TFS) are proteins that play important roles in complex biological processes, such as metabolism, autophagy, apoptosis, immune response, stem cell maintenance and differentiation [24].

The TCGA database of 163 cases of esophageal cancer has improved our ability to diagnose, treat, and prevent cancer [25]. Based on the mRNA expression matrix and clinical data from the TCGA-ESCA cohort, we identified six prognostic-associated ZNF genes that may be clinically valuable biomarkers. Patients with ESCA were divided into two subgroups with different survival outcomes based on a prognostic model of six ZNF family genes. We also established a risk score model to predict the prognoses of patients with ESCA based on these prognostic genes. Importantly, the ability of the prognostic model to distinguish high-and low-risk patients, and to estimate OS, was similarly validated in the GSE53624 dataset. Moreover, we combined risk score with other clinical variables to conduct a nomogram to establish a quantitative prognostic evaluation method for patients with ESCA.

Our ZNF family gene-based signature included six genes, i.e., ZNF91, ZNF586, ZNF502, ZNF865, ZNF106 and ZNF225. ZNF91 is likely to play an important role in cell proliferation and/or anti-apoptosis, and may serve as a molecular marker for AML [26]. Upregulation of ZNF91 could promote irradiation resistance by regulating the stem cell-like properties of NSCLC cells. Abnormal expression of ZNF91 is related to the occurrence and development of bladder cancer [26, 27], colorectal cancer (CRC) [28]and ovarian cancer [29, 30]. Our results also showed that ZNF91 is upregulated and plays an oncogenic role in ESCA. Genome-wide differential gene/microRNA signatures show that ZNF502 might be a prognostic biomarker in cytogenetically normal acute myeloid leukemia [31]. In our study, the expression of ZNF502 is low in esophageal carcinoma, which is associated with poor prognosis. ZNF106 is a RNA-binding protein that binds to the core splicing factor RNA-binding motif protein 39 and localizes to nuclear spots near the spliceosome [32]. We found that mRNA level of 106 was significantly reduced in ESCA tissues, this was similar to the reported results that ZNF106 expression was downregulated and associated with a good predictive value in Bladder Cancer [33, 34]. Little research has been done on the role of ZNF225 in ESCA, and a few evidences suggest ZNF225 inhibits autophagy and promotes apoptosis of hepatocellular carcinoma cells. Our findings on ZNF225 are supported by evidence that this ZNF protein serves as prognostic genes. In contrast, the roles of ZNF586, and ZNF865 in ESCA onset and development had not, to our knowledge, been as yet explored. Based on current knowledge, our findings suggest that the six prognosis-related ZNF family genes may exert important roles in the tumorigenesis and progression of ESCA.

By univariate Cox analysis, lasso regression and multivariate Cox analysis, we screened six prognosis-related ZNF family genes to construct the prognostic model. Survival and ROC curve analyses showed that these six genes had good diagnostic ability and could be used to screen out ESCA patients who had poor prognoses.when compared to previously reported models [35, 36], our model(At 1, 3, and 5 years, the risk score’s AUC values were 0.848, 0.872, and 0.952, respectively) has greater predictive power. However, the specific molecular mechanisms of these six prognosis-related ZNF family genes in ESCA remain unclear, and the underlying molecular mechanisms should be explored. Subsequently, we assessed the relationship between risk score model and clinical variables and found that the risk score model had significantly distinct risk stratification ability in ESCA. Nomogram has long been used in oncology to calculate the prognosis of patients with esophageal cancer based on the relevant clinical parameters [37–39]. We then established a nomogram to more intuitively predict 1-year, 3-year, and 5-year survival estimates in patients with ESCA and found that the risk score was more accurate than the pathological stage and age in predicting OS from TCGA and GEO dataset.

Next, we analyzed the differences in immune cell infiltration and response rates to chemotherapy sensitivity among different groups of patients with ESCA based on the model. ESCA was enriched in immune-suppressive cell populations, including Tregs, exhausted CD8 T, CD4 T and NK cells, M2 macrophages, and tDCs [40]. Accumulating evidence regards the tumor immune microenvironment can potentially influence the patient’s response to immune checkpoint inhibitors, tumor immunity, such as PD-L1 expression on tumors, tumor-infiltrating lymphocytes and tumor-associated macrophages [41]. Thus, our model can be used as an indicator to predict immune cell infiltration and immune response in patients with ESCA. At present, surgical resection, radiotherapy and chemotherapy are the main clinical treatment methods for ESCA, However, due to the limited efficacy and serious adverse effects of conventional treatment, the result is still unsatisfactory. As a new treatment method, target therapy has a good application prospect [41, 42]. In our study, patients in the high-risk group were more sensitive to AP-24,534, BMS-509,744, CGP-082996, HG-6-64-1, MG-132, Midostaurin, Ruxolitinib, Sunitinib, TAE684and Thapsigargin. From what has been discussed above, our results revealed differences in immune cell infiltration and immune response between the groups. ZNF-gene signature for ESCA was able to predict chemotherapy sensitivity and may thus help guide treatment selection.

We further conducted GO and KEGG analyses to evaluate biological functions. Enrichment analysis of biological functions and pathways of the ZNF family gene indicated that these prognosis gene were significantly enriched in Cell cycle, DNA replication and Fanconi anemia pathway.

Summarily, this study found that ZNF genes were differentially expressed in ESCA tissues and the reason may be different from the mechanism in the process of tumor formation. As the largest transcription regulator family in mammals, zinc finger (ZNF) protein expression regulation mechanism is very complex, including Genetic variation [43], Epigenetic modifications [44] and Posttranslational regulation [45]. We used the Kaplan-Meier analysis to study the prognostic significance of the six prognosis-related ZNF family genes and found that ZNF502, ZNF865, ZNF106 and ZNF225 gene expressions were related to good prognoses in patients with ESCA, while high ZNF586 and ZNF91 gene expressions were related to poor prognoses. We further confirmed the expressions of these genes at the tissue level. The results suggested that the signatures of these six genes may assess treatment outcomes and predict patient survival.

However, the current study has multiple limitations. Firstly, there are few normal tissues in TCGA database, which need to be verified by expanded samples. Second, the functional relationship between the ZNF gene signature members and non-tumor cells in the tumor microenvironment, especially infiltrating immune cells, could not be elucidated and requires future in vitro and in vivo studies. The effect on proliferation, invasion and migration of ZNF family genes in ESCA requires further be verified in vitro and in vivo.

## Conclusion

In summary, we first constructed a prognostic model of ESCA based on features of ZNF family genes that divides ESCA patients into two subgroups with different survival outcomes and constructed a nomogram to help clinical decision-makers provide optimal treatment. The prognostic signature is related to different immune cell and predicts sensitivity to chemotherapeutic agents which might be novel targets for developing immunotherapies in low-risk and high-risk ESCA patients. These prognosis-related ZNF family genes may play vital roles in ESCA occurrence, progression, invasion and metastasis. Additionally, these findings have led to the development of new clinical therapeutic targets or prognostic marker.

## Electronic supplementary material

Below is the link to the electronic supplementary material.

**Supplementary Table 1** Primer sequence

**Supplementary Table 3** Coefficients of six prognosis-related ZNF family genes in LASSO regression

**Supplementary Table 2** The clinical data of esophageal cancer patients in TCGA and GEO

Supplementary Material 4

Supplementary Material 5

Supplementary Material 6

Supplementary Material 7

Supplementary Material 8

Supplementary Material 9

## Data Availability

(ADM) The Supplementary Material for this article can be found online at this study, further inquiries can be directed to the corresponding authors. The datasets analyzed during the current study are available online from the GSE53624 dataset (https://www.ncbi.nlm.nih.gov/geo/query/acc.cgi?acc=GSE53624) and The Cancer Genome Atlas Program ().
