# Supplementary material for: Comprehensive analysis of ZNF family genes in prognosis, immunity, and treatment of esophageal cancer
Source: BMC Cancer. 2023 Apr 3;23:301. doi: 10.1186/s12885-023-10779-5 (PMC10069130; doi:10.1186/s12885-023-10779-5)
Supplement: Supplementary file 1 — Supplementary Table 1 Primer sequence [file 12885_2023_10779_MOESM1_ESM.docx]

| **Supplementary Table 1** Primer sequence | | |
| --- | --- | --- |
| Gene Name | Forward Primer | Reverse Primer |
| ZNF91 | TGAACCCACAGGTATATGTCCT | CGGATGCAAAATGACTTGACAC |
| ZNF502 | AGGAAGGAGGTTTTGGGAGAA | AGTGAGATGTGAGTGATTGCG |
| ZNF586 | CATTTCGCCGAAGCTCTTCAC | CCGCACTCATGCCTTTCTC |
| ZNF106 | AACCTTCCAATAGCAACCAAGAA | GCAGGCTGACTGTAACTCTCT |
| ZNF225 | GAGGAAACTGTACCGAGAAGTG | CAACCTGGCAGGGCATATCAT |
| ZNF865 | CTCGTGACTCACAAGTACGTG | GTGTAGGAGCAAGTCGTAGGC |
