# Supplementary material for: Comprehensive analysis of ZNF family genes in prognosis, immunity, and treatment of esophageal cancer
Source: BMC Cancer. 2023 Apr 3;23:301. doi: 10.1186/s12885-023-10779-5 (PMC10069130; doi:10.1186/s12885-023-10779-5)
Supplement: Supplementary file 2 — Supplementary Table 3 Coefficients of six prognosis-related ZNF family genes in LASSO regression [file 12885_2023_10779_MOESM2_ESM.docx]

| **Supplementary Table 3** Coefficients of six prognosis-related ZNF family genes in LASSO regression | |
| --- | --- |
| Gene Name | coefficients |
| ZNF91 | 0.852395788 |
| ZNF502 | -0.842766316 |
| ZNF586 | 1.926129889 |
| ZNF865 | -1.485081406 |
| ZNF106 | -1.016222299 |
| ZNF225 | -3.102275851 |
