# Supplementary material for: Comprehensive analysis of ZNF family genes in prognosis, immunity, and treatment of esophageal cancer
Source: BMC Cancer. 2023 Apr 3;23:301. doi: 10.1186/s12885-023-10779-5 (PMC10069130; doi:10.1186/s12885-023-10779-5)
Supplement: Supplementary file 3 — Supplementary Table 2 The clinical data of esophageal cancer patients in TCGA and GEO [file 12885_2023_10779_MOESM3_ESM.docx]

**Supplementary Table 2** The clinical data of esophageal cancer patients in TCGA and GEO

| TCGA  Characteristic | n (%) | GEO  Characteristic | n (%) |
| --- | --- | --- | --- |
| Age |  | Age |  |
| ≤60 | 83(50.9%) | ≤60 | 69 (58%) |
| >60 | 80(49.1%) | >60 | 50 (42%) |
| Gender |  | Gender |  |
| Female | 27 (14.8%) | Female | 21 (17.6%) |
| Male | 156 (85.2%) | Male | 98 (82.4%) |
| Stage |  | Stag |  |
| Stage I-II | 96 (60%) | stage I-II | 53 (44.5%) |
| Stage III-IV | 64 (40%) | stage III | 66 (55.5%) |
| Pathological TNM stage |  | Pathological TNM stage |  |
| T |  | T |  |
| T0-T2 | 75 (45.2%) | T1-T2 | 28 (23.5%) |
| T3-T4 | 91 (54.8%) | T3-T4 | 91 (76.5%) |
| N |  | N |  |
| N0-N1 | 144 (87.8%) | N0-N1 | 96 (80.7%) |
| N2-N3 | 20 (12.2%) | N2-N3 | 23 (19.3%) |
| M |  |  |  |
| M0 | 134 (93.7%) |  |  |
| M1 | 9 (6.3%) |  |  |
