# Supplementary figures and images for: Comprehensive analysis of ZNF family genes in prognosis, immunity, and treatment of esophageal cancer
Source: BMC Cancer. 2023 Apr 3;23:301. doi: 10.1186/s12885-023-10779-5 (PMC10069130; doi:10.1186/s12885-023-10779-5)

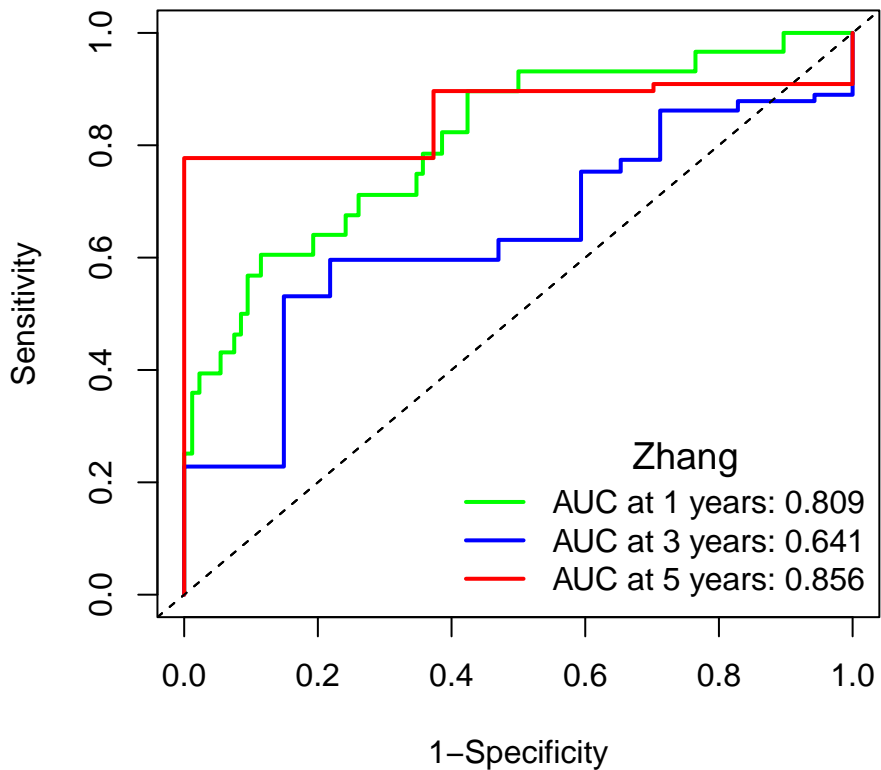

Supplement: Supplementary file 4 — Supplementary Material 4 [file 12885_2023_10779_MOESM4_ESM.pdf]

risk low high

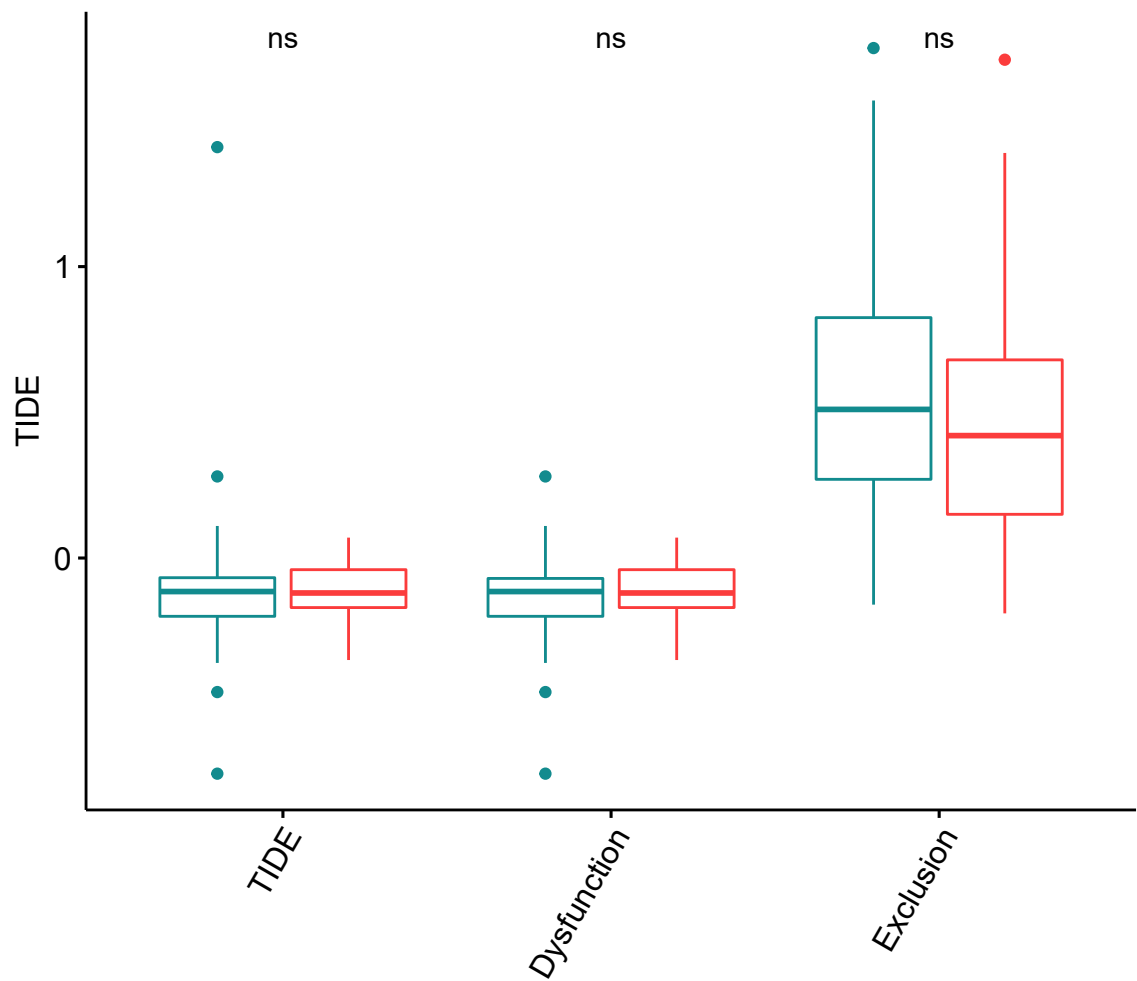

Supplement: Supplementary file 5 — Supplementary Material 5 [file 12885_2023_10779_MOESM5_ESM.pdf]

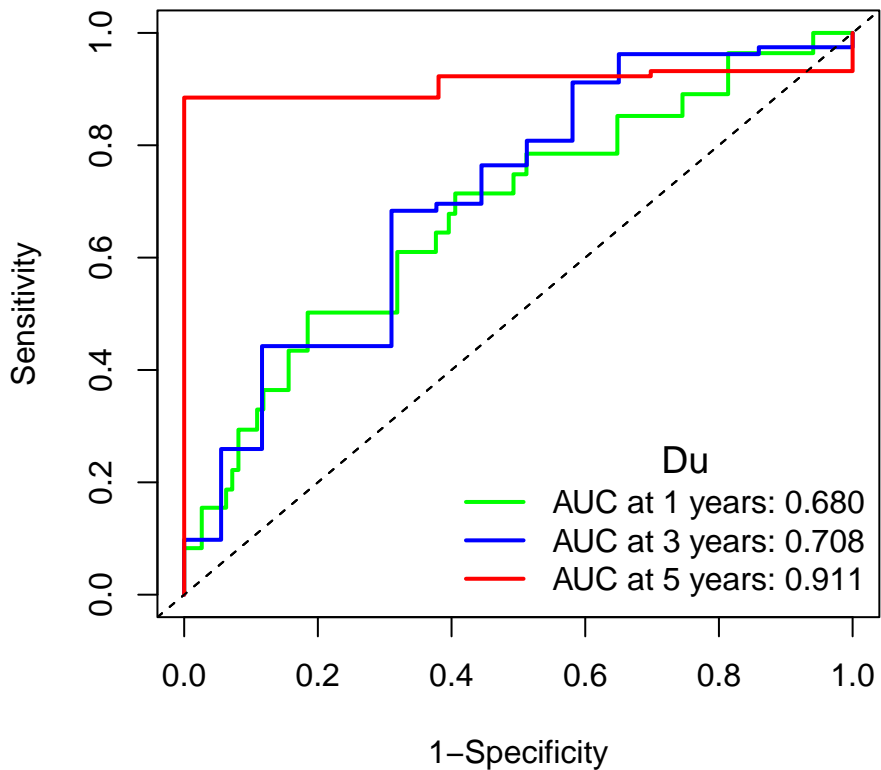

Supplement: Supplementary file 6 — Supplementary Material 6 [file 12885_2023_10779_MOESM6_ESM.pdf]
